# DUAL I China: Improved glycemic control with IDegLira versus its individual components in a randomized trial with Chinese participants with type 2 diabetes uncontrolled on oral antidiabetic drugs

**DOI:** 10.1111/1753-0407.13286

**Published:** 2022-06-28

**Authors:** Weiqing Wang, Bue F. Ross Agner, Bin Luo, Lei Liu, Ming Liu, Yongde Peng, Shen Qu, Karolina Amelia Stachlewska, Guixia Wang, Guoyue Yuan, Qiu Zhang, Guang Ning

**Affiliations:** ^1^ Department of Endocrine and Metabolic Diseases, Rui Jin Hospital Shanghai Jiao Tong University School of Medicine Shanghai China; ^2^ Bispebjerg Hospital Copenhagen Denmark; ^3^ Novo Nordisk China Pharmaceuticals Beijing China; ^4^ Novo Nordisk A/S Søborg Denmark; ^5^ Department of Endocrinology and Metabolism Tianjin Medical University General Hospital Tianjin China; ^6^ Department of Endocrinology and Metabolism, Shanghai General Hospital Shanghai Jiao Tong University Shanghai China; ^7^ Department of Endocrinology and Metabolism Shanghai Tenth People's Hospital of Tongji University Shanghai China; ^8^ Department of Endocrinology and Metabolism The First Hospital of Jilin University Jilin China; ^9^ Department of Endocrinology and Metabolism Affiliated Hospital of Jiangsu University Zhenjiang China; ^10^ Department of Endocrinology and Metabolism The First Affiliated Hospital of Anhui Medical University Hefei China

**Keywords:** Chinese, clinical trial, insulin, liraglutide, type 2 diabetes, 中国, 临床试验, 胰岛素, 利拉鲁肽, 2型糖尿病

## Abstract

**Background:**

DUAL I China, one of the DUAL trials, assessed efficacy/safety of insulin degludec/liraglutide (IDegLira) in Chinese adults with type 2 diabetes (T2D) not controlled by oral antidiabetic drugs (OADs).

**Methods:**

This phase 3a, treat‐to‐target multicenter trial randomized participants (glycated hemoglobin [HbA1c] 53.0‐85.8 mmol/mol; previous metformin ± another OAD) 2:1:1 to IDegLira (n = 361), degludec (n = 179), or liraglutide (n = 180). Primary endpoint was change in HbA1c after 26 weeks. Secondary endpoints included: HbA1c < 53.0 mmol/mol attainment, weight change, treatment‐emergent hypoglycemia, end‐of‐treatment insulin dose, and safety.

**Results:**

At 26 weeks, HbA1c had decreased by a mean 18.12 mmoL/moL (IDegLira), 12.37 mmoL/moL (degludec) (estimated treatment difference [ETD] −6.50 mmoL/moL; 95% confidence interval [CI] −7.96, −5.04; *P* < .0001), and 11.33 mmoL/moL (liraglutide) (ETD −6.87 mmoL/moL; 95% CI −8.33, −5.41; *P* < 0.0001), indicating noninferiority for IDegLira vs degludec and superiority vs liraglutide. HbA1c < 53.0 mmoL/moL attainment was 77.0% (IDegLira), 46.4% (degludec), and 48.3% (liraglutide). Mean weight change with IDegLira (0.1 kg) was superior to degludec (1.2 kg) (ETD −1.08 kg; 96% CI −1.55, −0.62; *P* < 0.0001). Severe or confirmed hypoglycemic event rates were 0.24 (IDegLira) and 0.17 (degludec) episodes/participant‐year (estimated rate ratio 1.46; 95% CI 0.71, 3.02; *P* = .3008, not significant). At the end of treatment, the IDegLira insulin dose was lower (24.5 U/d) vs degludec (30.3 U/d) (ETD −5.49 U; 95% CI −7.77, −3.21; *P* < 0.0001). No unexpected safety issues occurred.

**Conclusions:**

IDegLira is efficacious and well tolerated in Chinese adults with T2D not controlled by OADs.

## Graphical abstract

**Highlights**

- Insulin degludec/liraglutide (IDegLira) has good efficacy and tolerability in Chinese adults with type 2 diabetes not controlled on oral antidiabetic drugs.
- IDegLira provides the clinical advantages of insulin and glucagon‐like peptide 1 receptor agonists, improving glycemic control while reducing the main adverse effects of insulin (weight gain) and liraglutide (gastrointestinal events).
- As IDegLira is given as a once‐daily single injection, fewer injections are required versus administering the individual components separately.

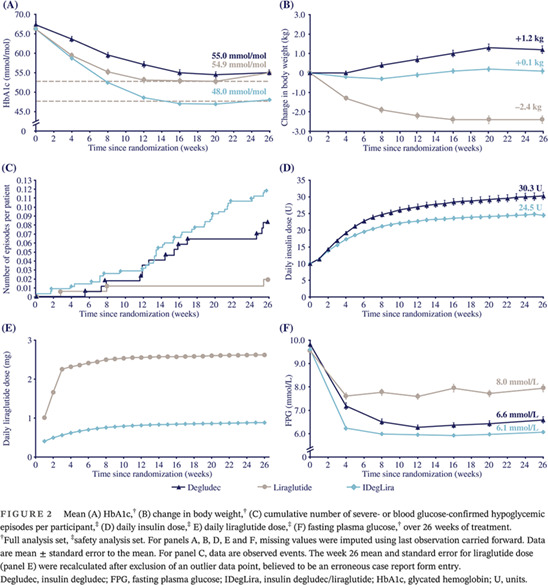

## 摘要

**背景:**

作为DUAL试验之一的DUAL I China评估了未口服抗糖药物(OADs)控制血糖的中国成人2型糖尿病(T2D)患者应用德谷胰岛素/利拉鲁肽(IDegLira)的有效性/安全性。

**方法:**

这项3a期, 治疗导向的多中心随机对照试验中, 受试者糖化血红蛋白(HbA1c)为53.0‐85.8 mmol/mol, 之前治疗为二甲双胍配合或不配合其他口服抗糖药物。按2:1:1的比例分别给予IDegLira(n=361), 德谷胰岛素(n=179)或利拉鲁肽(n=180)。主要终点是26周后HbA1c的变化。次要终点包括:HbA1c<53.0 mmol/mol, 体重变化, 治疗后出现的低血糖, 治疗结束时的胰岛素剂量, 以及安全性。

**结果:**

治疗26周时, 糖化血红蛋白平均下降18.12mmol/mol(IDegLira), 12.37mmol/mol(德谷胰岛素)(治疗前后差值估计(ETD)为‐6.50 mmoL/m ol, 95%可信区间−7.96, −5.04, P<0.0001)和11.33 mmoL/mol(利拉鲁肽)(ETD−6.87 mmoL/mol, 95%置信区间−8.33, −5.41, P<0.0001), 表明IDegLira相对于德谷胰岛素的非劣效性, 以及相对于利拉鲁肽的优越性。糖化血红蛋白(HbA1c)的达标率为77.0%(IDegLira), 46.4%(德谷胰岛素)和48.3%(利拉鲁肽)。平均体重变化:IDegLira组(0.1 kg)优于德谷胰岛素组(1.2 kg), ETD为‐1.08 kg(95%CI−1.5 5, −0.62), P<0.001。严重或确认的低血糖事件发生率分别为0.24(IDegLira)和0.17(德谷胰岛素)次/参与者‐年, 估计比率为1.46(95%CI:0.71, 3.02), P=.3008, 无显著意义。治疗结束时, IDegLira胰岛素剂量(24.5U/天)低于德谷胰岛素(30.3U/天), ETD为‐5.49U[95%CI:−7.77, −3.21], P<0.0001。没有发生意外安全事件。

**结论:**

IDegLira治疗非OADs控制的中国成人T2D是有效的且耐受性良好。

## 1 INTRODUCTION

In 2019, China had more people with type 2 diabetes (T2D) than any other nation, and this is projected to remain the case until at least 2045.^1^ This situation is likely to be due to an aging population and westernization of lifestyles.^2^ Compared with Caucasians, Asian people with T2D have different clinical features, such as a lower body mass index (BMI), greater abdominal adiposity, greater insulin resistance, and a younger age at onset.^3^ Consequently, Asian people with T2D may have differing responses to T2D therapies compared with Caucasians,^4^, ^5^, ^6^ necessitating confirmatory studies in Chinese participants.

Owing to disease progression, most people with T2D will eventually require treatment intensification. The Chinese Diabetes Society recommends a stepwise approach, progressing from lifestyle management to pharmacotherapy.^2^ These guidelines^2^ advise the addition of either glucagon‐like peptide 1 receptor agonists (GLP‐1RAs) or insulin to metformin when monotherapy is insufficient. If dual therapy fails, a triple combination is recommended (ie, metformin with GLP‐1RA and insulin) for Chinese individuals with T2D,^2^ and this is also supported by expert clinical opinion.^7^

Insulin degludec/liraglutide (IDegLira) is a subcutaneously administered, once‐daily combination therapy that contains insulin degludec (degludec) and the GLP‐1RA liraglutide in a fixed ratio (100 units/mL degludec:3.6 mg/mL liraglutide).^8^ The efficacy and safety of IDegLira were extensively investigated by the global DUAL trials, which demonstrated that IDegLira provides effective, enduring glycemic control with good tolerability in multinational participant populations.^8^, ^9^, ^10^, ^11^, ^12^, ^13^, ^14^, ^15^, ^16^ The global DUAL I trial enrolled participants with T2D who were not achieving their target glycated hemoglobin (HbA1c) levels with oral antidiabetic drugs (OADs) to investigate the efficacy of IDegLira versus its individual components.^8^ The trial demonstrated that improvement in HbA1c levels for IDegLira‐treated participants was noninferior versus degludec, and superior versus liraglutide. Compared with participants treated with degludec, IDegLira‐treated participants had lower weight gain and reduced rates of hypoglycemia, which have been associated with insulins such as degludec,^17^ as well as lower rates of gastrointestinal adverse events (AEs), which occur frequently with GLP‐1RAs.^18^ The DUAL I China trial (NCT03172494) aimed to confirm whether these benefits also apply to Chinese people with T2D that is not adequately controlled by OAD treatment.

## 2 METHODS

### 2.1 Trial design and participants

**FIGURE 1:**
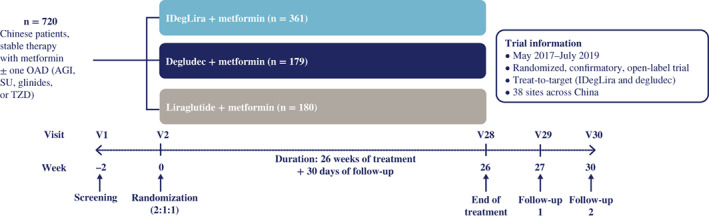
Trial design. AGI, alpha glucosidase inhibitor; degludec, insulin degludec; IDegLira, insulin degludec/liraglutide; OAD, oral antidiabetic drug; SU, sulphonylurea; TZD, thiazolidinedione; V, visit.

This phase 3a, 26‐week, multicenter, open‐label, randomized, parallel three‐arm, treat‐to‐target trial was of approximately 32 weeks' duration, with a 26‐week treatment period following a 2‐week screening period (Figure 1).

The protocol, information sheet, and consent form were approved after review by appropriate health authorities, independent institutional review boards, and ethics committees. The trial was conducted according to the guidelines of the International Conference on Harmonization Guideline for Good Clinical Practice^19^ and the Declaration of Helsinki.^20^ All participants gave informed, written consent.

The participants were Chinese people with T2D, aged ≥18 years, with HbA1c 53.0 to 85.8 mmoL/moL (7.0%‐10.0%), BMI ≤ 40 kg/m^2^, and on a stable dose of metformin (≥1500 mg or the maximum tolerated) ± one other OAD (α‐glucosidase inhibitor, sulfonylurea, glinide, or thiazolidinedione; at least half the local‐label maximum approved dose) for ≥60 days before screening.

Included in the key exclusion criteria were current treatment with other diabetic medications, previous insulin use (except short‐term treatment at investigator's discretion), and anticipated initiation/change in concomitant medications affecting glucose metabolism. Individuals with renal or liver impairment were excluded. **Supplementary Table** **1** lists full inclusion and exclusion criteria.

### 2.2 Stratification and randomization

Centralized randomization of participants was in a 2:1:1 ratio, following simple randomization procedures involving a web response system accessed via internet or telephone. Randomization was stratified by pretrial OADs (metformin ± one other OAD).

### 2.3 Treatment

After randomization, all participants continued metformin, and other OADs were discontinued. Participants initiated IDegLira at 10 dose steps (10 U degludec:0.36 mg liraglutide) and were titrated twice per week to a 4‐ to 5‐mmoL/L prebreakfast self‐measured plasma glucose (SMPG) target (**Supplementary Table** **2**). Degludec was started at 10 U and similarly titrated. IDegLira and degludec were administered in a similar treat‐to‐target approach; for IDegLira, the maximum daily dose given was 50 dose steps, but there was no maximum dose for degludec. Liraglutide was started at 0.6 mg and increased weekly by 0.6 mg, with the maximum target dose being 1.8 mg. If dose escalation was not tolerated, the visit window was used to prolong exposure time for a given dose (note, increments of less than 0.6 mg are not possible with the liraglutide administration pen device).

### 2.4 Endpoints

The primary endpoint was the week 26 change in HbA1c from baseline, aiming to confirm noninferiority for IDegLira versus degludec, along with superiority versus liraglutide.

Change in body weight after 26 weeks and the number of treatment‐emergent hypoglycemic episodes (severe according to the American Diabetes Association classification^21^ or confirmed by plasma glucose <3.1 mmoL/L [<56 mg/dL] with/without hypoglycemic symptoms) during the 26 weeks were confirmatory secondary endpoints, assessed with the aim of confirming superiority for IDegLira versus degludec.

Supportive secondary efficacy endpoints (assessed at week 26) included: change in fasting plasma glucose (FPG), nine‐point SMPG profile (including mean values across the nine‐point profile and the postprandial increments), attainment of HbA1c <53.0 mmoL/moL (7.0%) and ≤47.5 mmoL/moL (6.5%), HbA1c <53 mmoL/moL (7.0%) and ≤47.5 mmoL/ moL (6.5%) without weight gain and/or without severe or blood glucose (BG)‐confirmed hypoglycemic episodes during the final 12 weeks of treatment, total daily insulin dose, and fasting lipid profile. HbA1c and FPG were measured from blood samples in a central laboratory, while patients were issued with (and trained in using) a BG meter, which was used for the assessment of nine‐point SMPG profiles.

Safety endpoints included treatment‐emergent hypoglycemic events that were either severe or symptomatic and confirmed by BG measurement (overall and nocturnal events), as well as all treatment‐emergent AEs and vital signs.

### 2.5 Statistical analyses

For the primary endpoint (change in HbA1c from baseline after 26 weeks), the difference was compared with a margin for noninferiority of 0.4% for IDegLira versus degludec,^22^ and with a margin for superiority of 0.0% for IDegLira versus liraglutide. The sample size was determined by *t* statistic, assuming a one‐sided test of size 2.5% for both superiority and noninferiority; the mean between‐treatment difference in the HbA1c change (baseline to 26 weeks) was assumed to be 0%‐points for noninferiority and −0.3%‐points for superiority testing, both with a standard deviation of 1.0%. The per‐protocol analysis set (assumed as 85% of those randomized) was used to calculate sample size for noninferiority evaluation and the full analysis set for superiority evaluation. These assumptions indicated that a sample size of n = 720 would result in a power of 98.1% for noninferiority and a 90.7% power for superiority, with a power for reaching the primary objective of 89.0%.

A hierarchical testing procedure was applied to control the overall type I error rate based on an a priori ordering of the null hypotheses. An analysis of covariance (ANCOVA) model was used to assess the primary endpoint (treatment and previous OAD as fixed factors; baseline HbA1c as a covariate), with missing values imputed using “last observation carried forward.” Continuous secondary endpoints were analyzed with an ANCOVA model (with the same fixed factors as for the primary endpoint; respective baseline value as covariate; for insulin dose: baseline HbA1c as covariate; for fasting lipids: endpoint and baseline value log‐transformed). The nine‐point SMPG profile was analyzed by a linear mixed‐effects model. Attainment of HbA1c targets was analyzed by a logistic regression model. The number of treatment‐emergent severe or confirmed hypoglycemic events was analyzed by negative binominal regression using a log‐link function with logarithm of the exposure time as offset and with the same fixed factors as for the primary endpoint.

## 3 RESULTS

### 3.1 Participants

Recruitment took place between May 2017 and December 2018 from 38 sites in mainland China. Overall, 720 participants were randomized to IDegLira (n = 361), degludec (n = 179), or liraglutide (n = 180) (**Supplementary Figure** **1**), and 713 received study drugs (n = 358 IDegLira, n = 175 degludec, and n = 180 liraglutide). Sixty‐one participants withdrew: 20 (5.5%), 12 (6.7%), and 29 (16.1%), respectively, from the IDegLira, degludec, and liraglutide groups. Withdrawals resulting from AEs occurred at a greater rate in the liraglutide group (8.3% [n = 15]) versus IDegLira (1.4% [n = 5]) and degludec (0.6% [n = 1]).

Baseline characteristics (Table 1) were similar across groups. Approximately one‐third (32.5%) of participants were treated with metformin only. The most common combined OAD regimen at screening was metformin and sulfonylureas (38.1%), followed by metformin combined with α‐glucosidase inhibitors (20.1%).

**TABLE 1:** Baseline characteristics (full analysis set)

Characteristic	IDegLira (n = 361)	Degludec (n = 179)	Liraglutide (n = 180)	Total (N = 720)
Age, y	54.5 (10.3)	55.7 (10.2)	54.1 (10.2)	54.7 (10.3)
Sex, n (%)				
Female	142 (39.3)	79 (44.1)	72 (40.0)	293 (40.7)
Male	219 (60.7)	100 (55.9)	108 (60.0)	427 (59.3)
Weight, kg	74.8 (14.3)	73.2 (13.2)	73.8 (13.3)	74.1 (13.8)
BMI, kg/m^2^	27.0 (3.9)	26.5 (3.6)	26.5 (3.6)	26.7 (3.7)
Duration of diabetes, y	8.00 (5.33)	8.63 (5.55)	8.05 (5.28)	8.17 (5.37)
HbA1c, %	8.20 (0.83)	8.31 (0.84)	8.21 (0.77)	8.23 (0.82)
HbA1c, mmol/mol	66.17 (9.06)	67.34 (9.17)	66.27 (8.44)	66.48 (8.94)
Fasting plasma glucose, mmol/L	9.61 (2.03)	9.83 (2.07)	9.55 (2.01)	9.65 (2.04)
Fasting plasma glucose, mg/dL	173.09 (36.67)	177.13 (37.35)	172.07 (36.28)	173.84 (36.74)
OAD at screening, n (%)				
Metformin only	118 (32.7)	57 (31.8)	59 (32.8)	234 (32.5)
Metformin + sulfonylureas	137 (38.0)	71 (39.7)	66 (36.7)	274 (38.1)
Metformin + α‐glucosidase inhibitors	74 (20.5)	34 (19.0)	37 (20.6)	145 (20.1)
Metformin + glinides	20 (5.5)	13 (7.3)	12 (6.7)	45 (6.3)
Metformin + thiazolidinediones	12 (3.3)	4 (2.2)	6 (3.3)	22 (3.1)

*Note:* Data are mean (SD) unless otherwise stated. Baseline refers to week 0, except for BMI, which was taken at screening (week −2). The duration of diabetes was calculated as the time from date of diagnosis to the randomization date.

Abbreviations: BMI, body mass index; degludec, insulin degludec; HbA1c, glycated hemoglobin; IDegLira, insulin degludec/liraglutide; OAD, oral antidiabetic drug; SD, standard deviation.

### 3.2 Glycated hemoglobin

**FIGURE 2:**
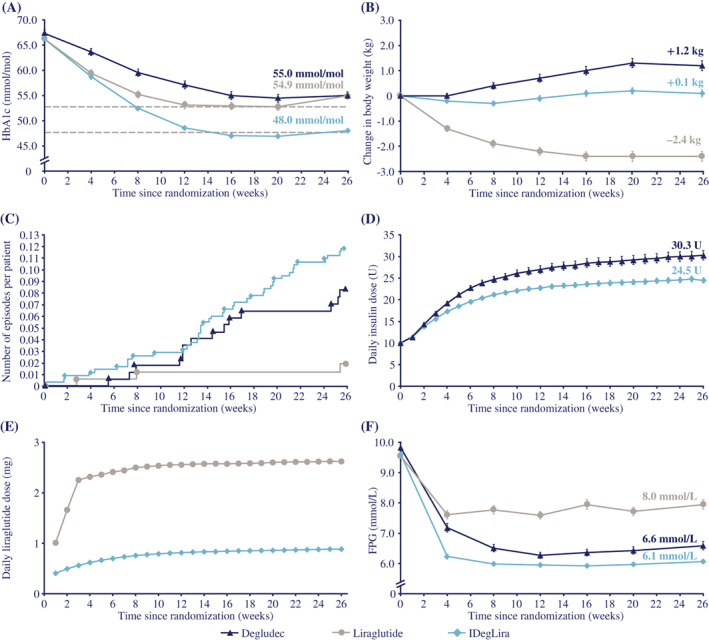
Mean (A) HbA1c,^†^ (B) change in body weight,^†^ (C) cumulative number of severe‐ or blood glucose‐confirmed hypoglycemic episodes per participant,^‡^ (D) daily insulin dose,^‡^ E) daily liraglutide dose,^‡^ (F) fasting plasma glucose,^†^ over 26 weeks of treatment. ^†^Full analysis set, ^‡^safety analysis set. For panels A, B, D, E and F, missing values were imputed using last observation carried forward. Data are mean ± standard error to the mean. For panel C, data are observed events. The week 26 mean and standard error for liraglutide dose (panel E) were recalculated after exclusion of an outlier data point, believed to be an erroneous case report form entry. Degludec, insulin degludec; FPG, fasting plasma glucose; IDegLira, insulin degludec/liraglutide; HbA1c, glycated hemoglobin; U, units.

After 26 weeks, HbA1c had decreased from the baseline mean of 66.17 mmoL/moL (8.20%) to 48.04 mmoL/moL (6.55%) with IDegLira and from 67.34 mmoL/moL (8.31%) to 54.97 mmoL/moL (7.18%) with degludec: reductions of 18.12 mmoL/moL (1.66%) versus 12.37 mmoL/moL (1.13%), respectively. The HbA1c reduction was statistically significantly greater with IDegLira versus degludec (estimated treatment difference [ETD] –6.50 mmoL/moL [−0.59%]; 95% CI −7.96, −5.04; *P* < 0.0001), hence noninferiority of IDegLira was confirmed (Figure 2A). There was a mean decrease in HbA1c from 66.27 mmoL/moL (8.21%) to 54.94 mmoL/moL (7.18%) with liraglutide (11.33 mmoL/moL [1.04%] reduction), demonstrating IDegLira superiority versus liraglutide (ETD –6.87 mmoL/moL [−0.63%]; 95% CI −8.33, −5.41; *P* < 0.0001) (Figure 2A).

### 3.3 Body weight

After 26 weeks, there was a lesser gain in body weight with IDegLira (mean increase 0.1 kg) versus degludec (mean increase 1.2 kg), confirming the superiority of IDegLira to degludec (ETD −1.08 kg; 95% CI −1.55, −0.62; *P* < 0.0001) (Figure 2B). The body weight change was statistically significantly different in favor of liraglutide (mean 2.4 kg reduction) versus IDegLira (ETD 2.57; 95% CI 2.10, 3.04; *P* < 0.0001) (Figure 2B).

### 3.4 Hypoglycemia

The rates of treatment‐emergent severe or confirmed hypoglycemic episodes were 0.24 versus 0.17 episodes/participant‐year of exposure (PYE) with IDegLira and degludec, respectively (Table 2 **;** Figure 2C). Superiority of IDegLira was not found: estimated rate ratio (ERR) 1.46; 95% CI 0.71, 3.02; *P* = 0.3008 (not statistically significant). A statistically significantly lower rate of treatment‐emergent severe or confirmed hypoglycemic events occurred with liraglutide (0.04 events/PYE) versus IDegLira (Table 2 **;** Figure 2C) (ERR 6.36; 95% CI 1.83, 22.15; *P* = 0.0036). The proportions of participants experiencing at least one episode were similar in both insulin groups (IDegLira: 8.1%; degludec: 8.0%) and lower with liraglutide (1.7%). Nocturnal treatment‐emergent severe or confirmed hypoglycemic episodes are shown in Table 2.

**TABLE 2:** Treatment‐emergent hypoglycemic episodes (safety analysis set)

Events	IDegLira (n = 358)				Degludec (n = 175)				Liraglutide (n = 180)			
	N	%	E	R	N	%	E	R	N	%	E	R
**Number of participants**	358				175				180			
Participant‐years of exposure	179.6				88.2				83.4			
Hypoglycemic episodes												
Severe or BG‐confirmed symptomatic hypoglycemia	20	5.6	27	0.15	8	4.6	8	0.09	1	0.6	1	0.01
Severe or BG‐confirmed hypoglycemia	29	8.1	43	0.24	14	8.0	15	0.17	3	1.7	3	0.04
Nocturnal hypoglycemic episodes												
Severe or BG‐confirmed symptomatic hypoglycemia	5	1.4	5	0.03	3	1.7	3	0.03	0	0	0	0
Severe or BG‐confirmed hypoglycemia	6	1.7	8	0.04	4	2.3	4	0.05	0	0	0	0

Abbreviations: %, percentage of participants with one or more events; BG, blood glucose; degludec, insulin degludec; E, number of events; IDegLira, insulin degludec/liraglutide; N, number of participants with one or more events; R, rate (number of events divided by participant‐years of exposure).

### 3.5 Insulin and liraglutide dose

After 26 weeks, participants receiving IDegLira had a statistically significantly lower mean daily insulin dose (24.5 U) versus degludec‐treated participants (30.3 U) (ETD −5.49 U; 95% CI −7.77, −3.21; *P* < 0.0001; Figure 2D). The lower insulin doses (U/kg) with IDegLira were consistent when body weight was accounted for (**Supplementary Table 3**). Participants receiving IDegLira had a lower liraglutide dose (0.9 mg) at week 26 versus those receiving liraglutide (1.8 mg) (Figure 2E).

### 3.6 Fasting plasma glucose

IDegLira treatment provided a statistically significantly greater reduction from baseline in FPG versus degludec (−3.51 vs. −3.24 mmoL/L; ETD −0.47 mmoL/L; 95% CI −0.76, −0.17; *P* = 0.0021) and liraglutide (−3.51 vs. −1.60 mmoL/L; ETD −1.87 mmoL/L; 95% CI −2.16, −1.57; *P* < 0.0001) (Figure 2F).

### 3.7 Self‐measured plasma glucose

**FIGURE 3:**
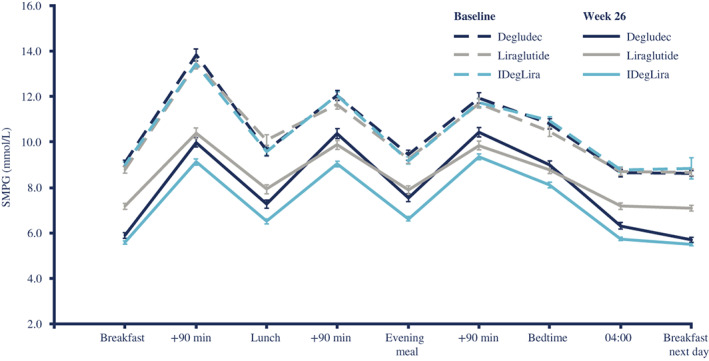
Nine‐point SMPG profile (full analysis set) Degludec, insulin degludec; IDegLira, insulin degludec/liraglutide; SMPG, self‐measured plasma glucose.

The nine‐point SMPG decreased (at all time points) from baseline in all groups over 26 weeks (Figure 3). IDegLira showed statistically significantly lower SMPG values at all time points, except before breakfast and before breakfast next day, versus degludec and at all time points versus liraglutide. IDegLira provided a statistically significantly greater 26‐week reduction in the mean nine‐point SMPG profile versus degludec (ETD −0.78 mmoL/L; 95% CI −1.06, −0.51; *P* < 0.0001), and liraglutide (ETD −1.12 mmoL/L; 95% CI −1.40, −0.85; *P* < 0.0001).

The reduction in postprandial glucose increments was statistically significantly greater with IDegLira versus degludec at breakfast time (ETD −0.47 mmoL/L; 95% CI −0.91, −0.03; *P* = 0.0359), lunch (−0.55 mmoL/L; 95% CI −1.00, −0.10; *P* = 0.0176), and for the mean of all meals (−0.41 mmoL/L; 95% CI −0.70, −0.12; *P* = 0.0055), but a statistically significant difference was not seen for the evening meal (−0.09 mmoL/L; 95% CI −0.54, 0.36; *P* = 0.6930). A statistically significantly greater reduction in postprandial glucose increments was observed with liraglutide versus IDegLira for the evening meal (ETD 0.76 mmoL/L; 95% CI 0.31, 1.22; *P* = 0.0010) and for the mean of all meals (0.44 mmoL/L; 95% CI 0.15, 0.73; *P* = 0.0029); however, the differences at breakfast (0.31 mmoL/L; 95% CI –0.12, 0.75; *P* = 0.1604) and lunch (0.38 mmoL/L; 95% CI −0.07, 0.83; *P* = 0.1015) were not statistically significant.

### 3.8 HbA1c responders

**FIGURE 4:**
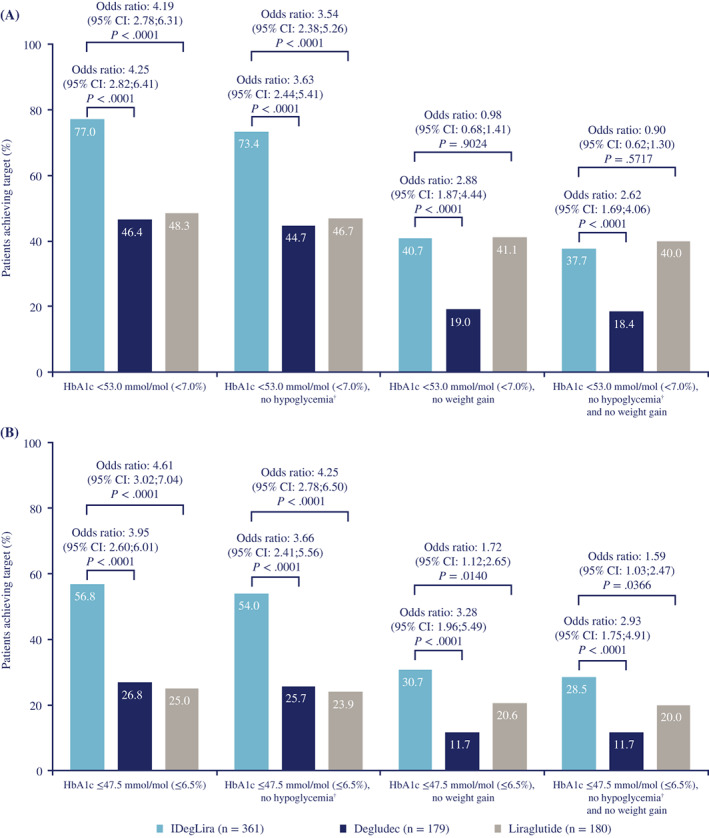
Responder endpoints for (A) HbA1c <53.0 mmoL/moL, and (B) HbA1c ≤48.0 mmoL/moL, and composite endpoints for reaching these targets without weight gain and/or without treatment‐emergent severe or confirmed hypoglycemic episodes^†^ (full analysis set) ^†^Treatment‐emergent severe or BG‐confirmed hypoglycemic episodes during the final 12 weeks of treatment. Percentages are observed data. Missing values were imputed using last observation carried forward. Analysis after 26 weeks of treatment based on a logistic regression model with treatment and previous oral antidiabetic treatment as fixed factors. The covariate for: ‘Responder for HbA1c <7.0% and for HbA1c ≤6.5%’, and for ‘HbA1c responder endpoints without hypoglycemic episodes’ was baseline HbA1c; covariates for: ‘HbA1c responder endpoints without weight gain’, and for ‘HbA1c responder endpoints without hypoglycemic episodes and weight gain’ were baseline HbA1c and body weight. BG, blood glucose; CI, confidence interval; degludec, insulin degludec; IDegLira, insulin degludec/liraglutide.

The respective proportions of participants attaining HbA1c <53 mmoL/moL (7.0%) with IDegLira, degludec, and liraglutide were 77.0%, 46.4%, and 48.3%. There were statistically significantly greater odds of attaining HbA1c <53.0 mmoL/moL (7.0%) with IDegLira than with degludec (*P* < 0.0001) and liraglutide (*P* < 0.0001) (Figure 4A). The respective proportions of participants attaining HbA1c ≤47.5 mmoL/moL (6.5%) with IDegLira, degludec, and liraglutide were 56.8%, 26.8%, and 25.0%. Again, the odds of attaining this target were statistically significantly greater with IDegLira than with degludec (*P* < 0.0001) and liraglutide (*P* < 0.0001) (Figure 4B).

Of participants receiving IDegLira, 338 received <50 dose steps at week 26, and 267 of these reached the HbA1c target <53.0 mmoL/moL (7.0%), giving a responder rate of 79.0%. There were 18 participants receiving IDegLira who titrated to the dose limit of 50 dose steps (5% of the IDegLira group); of these, 11 (61.1%) reached the HbA1c target <53.0 mmoL/moL (7.0%).

### 3.9 Composite responder endpoints

Figure 4 shows composite responder endpoints for reaching the HbA1c targets without gaining weight and/or without experiencing severe or confirmed hypoglycemia. The odds of achieving these triple composite responder endpoints (for both HbA1c targets) were statistically significantly greater with IDegLira versus degludec (both *P* < 0.0001).

There was no statistically significant difference between IDegLira and liraglutide for achievement of the triple composite endpoint including the HbA1c target of <53.0 mmoL/moL (7.0%) (*P* = 0.5717), but the difference was statistically significant for the triple composite endpoint including the HbA1c target of ≤47.5 mmoL/moL (6.5%) (*P* = 0.0366).

### 3.10 Other outcomes

Vital signs and lipid profiles are reported in **Supplementary Table** **4** and **Supplementary Table** **5**, respectively.

### 3.11 Adverse events

The proportions of participants with reported AEs and the AE rates were higher in those receiving GLP‐1RAs; 75% (4.11 events/PYE) experienced an AE with IDegLira, 79% (5.41 events/PYE) with liraglutide, and 67% (3.06 events/PYE) with degludec (Table 3). The majority of AEs were of mild‐to‐moderate severity and, other than gastrointestinal AEs, most were thought as being unlikely related to treatment. Table 3 lists the AEs most frequently reported. Gastrointestinal events were reported by 21.2% of participants receiving IDegLira, which was lower versus liraglutide (42.2%) and higher versus degludec (9.7%); most common were diarrhea and nausea.

**TABLE 3:** Treatment‐emergent adverse events (safety analysis set)

Events	IDegLira (n = 358)				Degludec (n = 175)				Liraglutide (n = 180)			
	N	%	E	R	N	%	E	R	N	%	E	R
**AEs**	267	74.6	738	4.11	117	66.9	270	3.06	143	79.4	451	5.41
AEs possibly or probably related to treatment	107	29.9	187	1.04	18	10.3	23	0.26	101	56.1	210	2.52
**Most frequent AE (≥5% of participants)**												
Infections and infestations												
Viral upper respiratory tract infection	66	18.4	83	0.46	31	17.7	34	0.39	18	10	20	0.24
Nasopharyngitis	16	4.5	20	0.11	9	5.1	11	0.12	5	2.8	7	0.08
Gastrointestinal disorders												
Diarrhea	23	6.4	31	0.17	3	1.7	3	0.03	26	14.4	41	0.49
Nausea	14	3.9	25	0.14	1	0.6	1	0.01	21	11.7	23	0.28
Abdominal discomfort	1	0.3	1	0.01	0	0	0	0	9	5.0	9	0.11
Gastrointestinal disorder	1	0.3	1	0.01	0	0	0	0	10	5.6	11	0.13
Metabolism and nutrition												
Hyperlipidemia	16	4.5	16	0.09	6	3.4	6	0.07	10	5.6	11	0.13
Decreased appetite	13	3.6	13	0.07	2	1.1	2	0.02	26	14.4	27	0.32
Eye disorders												
Diabetic retinopathy	28	7.8	28	0.16	6	3.4	6	0.07	10	5.6	10	0.12
Investigations												
Lipase increased	27	7.5	30	0.17	5	2.9	6	0.07	22	12.2	22	0.26
**SAEs**	14	3.9	17	0.09	7	4.0	8	0.09	14	7.8	14	0.17
SAEs possibly or probably related to treatment	1	0.3	1	0.01	1	0.6	1	0.01	1	0.6	1	0.01

*Note:* A treatment‐emergent event was an event occurring on or after the first day of trial product administration and no later than 7 days after the last day on trial product. Table includes preferred terms with ≥5% participants in at least one treatment group.

Abbreviations: %, percentage of participants with one or more events; AE, adverse event; degludec, insulin degludec; E, number of adverse events; IDegLira, insulin degludec/liraglutide; N, number of participants with one or more events; R, rate (number of adverse events divided by participant‐years of exposure); SAE, serious adverse event.

Twenty‐three participants experienced 35 AEs leading to dose reduction; IDegLira: 21 AEs in 13 participants, degludec: 6 AEs in 5 participants, and liraglutide: 8 AEs in 5 participants. In total, 25 AEs led to the withdrawal of 21 participants: 5, 1, and 19 AEs for IDegLira, degludec, and liraglutide, respectively.

Among adjudicated treatment‐emergent events, six were confirmed as major adverse cardiovascular events, all ischemic strokes that the investigators evaluated as being unlikely related to trial product. Two of these stroke events occurred with IDegLira and four with liraglutide; five of the stroke events were classed as serious. Adjudication confirmed two treatment‐emergent neoplasms with liraglutide (large intestinal polypectomy and cervical dysplasia). No pancreatitis events with any treatment were confirmed by adjudication.

The event rate/PYE of elevated amylase or lipase was 0.24, 0.12, and 0.32, respectively, with IDegLira, degludec, and liraglutide. The event rate/PYE of thyroid AEs was low with IDegLira (0.02), degludec (0.03), and liraglutide (0.05). There was one event of elevated calcitonin in each group (three in total).

### 3.12 Serious adverse events

The proportion of participants experiencing serious adverse events (SAEs) (and the rates of SAEs) were similar for IDegLira (3.9%; 0.09 events/PYE) and degludec (4.0%; 0.09 events/PYE) and higher with liraglutide (7.8%; 0.17 events/PYE) (Table 3). No SAEs were reported in ≥5% of patients. Three SAEs were judged as being possibly related to treatment by the investigator: gastroesophageal reflux disease (IDegLira), angina unstable (degludec), and arteriosclerosis coronary artery (liraglutide); no SAEs were judged as probably related to treatment. Two SAEs, gastroesophageal reflux disease (IDegLira) and angina unstable (degludec), led to dose reduction. Four SAEs led to permanent treatment discontinuation: lung neoplasm malignant (IDegLira), cardiac failure chronic (IDegLira), diabetes mellitus inadequate control (degludec), and cerebral infarction (liraglutide). There were no deaths reported in this study.

## 4 DISCUSSION

This treat‐to‐target trial in Chinese participants with T2D found noninferiority for IDegLira versus degludec and superiority versus liraglutide for improving HbA1c. This improvement was also reflected in the statistically significantly higher odds of attaining the two HbA1c targets after 26 weeks with IDegLira versus degludec and liraglutide. These results in Chinese participants were consistent with those of the global DUAL I trial.^8^ Compared with degludec, superiority of IDegLira for change in body weight was also confirmed in the present study in Chinese participants, although superiority was not confirmed for the number of treatment‐emergent severe or confirmed hypoglycemic events.

Due to the synergy between combined basal insulin and GLP‐1RA,^8^ glycemic control was achieved using lower doses of insulin and liraglutide with IDegLira than with either of the individual treatments alone. The additional reductions in postprandial plasma glucose increments by the liraglutide component appear to contribute to the greater HbA1c reduction seen with IDegLira versus degludec. Interestingly, laboratory‐measured mean FPG was also statistically significantly lower with IDegLira than with degludec treatment, and this would also contribute to a lower HbA1c. This difference might be regarded as unexpected, given that both IDegLira and degludec were titrated to the same SMPG target (**Supplementary Table** **2**). No statistically significant difference was observed, however, in the prebreakfast SMPG level from the nine‐point profiles. This apparent discrepancy (between the prebreakfast SMPG and FPG data) might reflect the different assay methodologies used or differences in sampling times.

IDegLira is considered weight‐neutral,^8^ supported by data from the global DUAL I trial and also from this Chinese trial. Body weight was maintained with IDegLira and within the range of previous clinical trials reporting fixed‐ratio combination therapies,^23^ in contrast with the weight gain among degludec‐treated participants. The modest differences in weight change may be due to lower body weight at baseline and the lower doses of IDegLira used in the China trial versus the global DUAL I trial.

We found a numerically higher rate (not statistically significant) of severe or confirmed hypoglycemia with IDegLira versus degludec, whereas a statistically significant lower rate of confirmed hypoglycemia with IDegLira than degludec was shown in the global DUAL I trial.^8^ Rates of severe or confirmed hypoglycemia, however, were low with both IDegLira and degludec in the China study relative to the global study, and this might have influenced these findings. Liraglutide is associated with low risk of hypoglycemia (this attributed to its action being glucose‐dependent^23^, ^24^), whereas hypoglycemia is a well‐recognized risk with insulin therapy.^17^ Thus, in both global and Chinese populations, liraglutide was associated with statistically significantly lower hypoglycemia rates versus IDegLira.

IDegLira reduced levels of total cholesterol after 26 weeks versus degludec and liraglutide, with reductions in low‐density lipoprotein versus degludec and lower levels of free fatty acids versus liraglutide. People with T2D frequently have raised plasma cholesterol and abnormalities of other plasma lipoproteins.^25^ Increased total cholesterol or low‐density lipoprotein are major risk factors for coronary heart disease,^26^ while changes in plasma and islet cholesterol metabolism may contribute to the pathogenesis of T2D.^25^ Although there is no direct evidence of a cardiovascular benefit of IDegLira, liraglutide has been demonstrated to provide cardiovascular benefits in patients with T2D^27^, ^28^; in the LEADER trial, occurrences of major cardiovascular events and cardiovascular deaths were lower among patients with T2D receiving liraglutide versus placebo.^27^ Insulin degludec, meanwhile, has also been shown to have equivalent cardiovascular safety when compared with insulin glargine.^29^

This study did not identify any unexpected safety issues, with the safety profile of IDegLira reflecting those of its components degludec^30^ and liraglutide.^31^ Gastrointestinal AEs, particularly diarrhea and nausea, occurred at lower rates with IDegLira versus liraglutide, as also observed in the global DUAL I trial.^8^ This is likely attributable to the lower starting dose of liraglutide in the IDegLira group, followed by slower increases in liraglutide dose as a component of IDegLira versus the liraglutide group.

A limitation of DUAL I China was the open‐label design; however, this was necessary because of the different methods of drug titration to a therapeutic dose—IDegLira and degludec both followed a titration algorithm, whereas liraglutide used dose escalation—and because IDegLira has a maximum dose limitation, whereas degludec can be continually uptitrated as required. However, the randomized controlled trial design and the large participant cohort can be considered strengths of this study.

In conclusion, the DUAL I China trial demonstrated that IDegLira is efficacious and well‐tolerated in Chinese people with T2D for whom the glycemic target cannot be met with OADs. Our findings are consistent with data from the global DUAL I trial. Either basal insulin or GLP‐1RA is recommended as intensification strategies for people whose T2D cannot be adequately controlled by OADs.^32^ IDegLira provides the clinical benefits of both insulin and GLP‐1RA but offers improved glycemic control while at the same time it reduces some of the key adverse effects of both insulin (eg, weight gain) and GLP‐1RAs (eg, adverse gastrointestinal events). Moreover, IDegLira, as a fixed‐ratio combination, provides degludec and liraglutide in a once‐daily injection, thereby reducing the number of injections versus administering the individual components. The benefits of combined treatment with IDegLira may help to counteract inertia around intensifying therapy when treatment targets are not achieved.

## DISCLOSURE

B.A., B.L., L.L., and K.S. are, or have been, employees of Novo Nordisk who own(ed) shares in the company during the time of manuscript development. W.W., M.L., Y.P., S.Q., G.W., G.Y., Q.Z., and G.N. declare no conflicts of interest. G.N. is an editor for the *Journal of Diabetes*.

## AUTHOR CONTRIBUTIONS

The authors declare that they each meet the International Committee of Medical Journal Editors (ICMJE) uniform requirements for authorship. They have contributed to data collection, to data analysis and interpretation, and to drafting and critically revising this article. They share final responsibility for submitting the article for publication and for its final content. W.W. accepts responsibility as guarantor for the work and the integrity of the data, having had full access to all study data.

## PRIOR PUBLICATION

Parts of this study were included in a poster presentation at the European Association for the Study of Diabetes, 56th Annual Meeting, 22 to 25 September 2020, Virtual Meeting.

## Supporting information


**Appendix S1** Supporting Information
