# Corrigendum

**DOI:** 10.1111/1753-0407.13307

**Published:** 2022-09-26

The authors would like to draw the reader’s attention to the updates in Abstract section, Figure 2E and the description of this panel in the Results section, and Figure 4 caption, for the following article:

Wang W, Agner BFR, Luo B, et al. DUAL I China: Improved glycemic control with IDegLira versus its individual components in a randomized trial with Chinese participants with type 2 diabetes uncontrolled on oral antidiabetic drugs. *Journal of Diabetes*. 2022; 14(6):401‐413. doi:10.1111/1753‐0407.13286

In Abstract, Results section, ‘18.12 mmoL/moL’ should read as ‘18.12 mmol/mol’ and the value ‘96% CI −1.55’ has been updated to ‘95% CI −1.55’. The section is shown below:

**Results:** At 26 weeks, HbA1c had decreased by a mean 18.12 mmol/mol (IDegLira), 12.37 mmoL/moL (degludec) (estimated treatment difference [ETD] −6.50 mmoL/moL; 95% confidence interval [CI] −7.96, −5.04; *P* < .0001), and 11.33 mmoL/moL (liraglutide) (ETD −6.87 mmoL/moL; 95% CI −8.33, −5.41; *P* < 0.0001), indicating noninferiority for IDegLira vs degludec and superiority vs liraglutide. HbA1c < 53.0 mmoL/moL attainment was 77.0% (IDegLira), 46.4% (degludec), and 48.3% (liraglutide). Mean weight change with IDegLira (0.1 kg) was superior to degludec (1.2 kg) (ETD −1.08 kg; 95% CI −1.55, −0.62; *P* < 0.0001). Severe or confirmed hypoglycemic event rates were 0.24 (IDegLira) and 0.17 (degludec) episodes/participant‐year (estimated rate ratio 1.46; 95% CI 0.71, 3.02; *P* = .3008, not significant). At the end of treatment, the IDegLira insulin dose was lower (24.5 U/d) vs degludec (30.3 U/d) (ETD −5.49 U; 95% CI −7.77, −3.21; *P* < 0.0001). No unexpected safety issues occurred.

The revised Figure 2 and caption are shown below:

**FIGURE 2:**
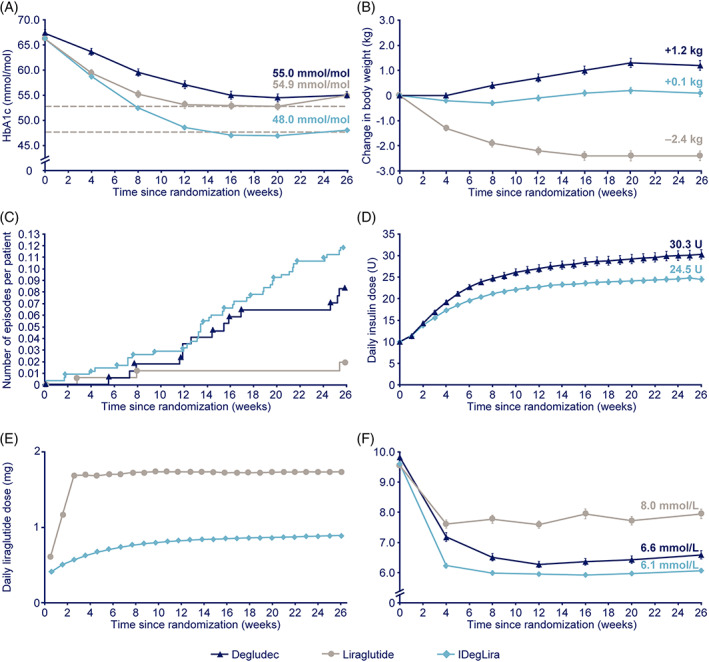
Mean (A) HbA1c,^†^ (B) change in body weight,^†^ (C) cumulative number of severe‐ or blood glucose‐confirmed hypoglycemic episodes per participant,^‡^ (D) daily insulin dose,^‡^ E) daily liraglutide dose,^‡^ (F) fasting plasma glucose,^†^ over 26 weeks of treatment. †Full analysis set, ^‡^safety analysis set. For panels A, B, D, E and F, missing values were imputed using last observation carried forward. Data are mean ± standard error to the mean. For panel C, data are observed events. The week 26 mean and standard error for liraglutide dose (panel E) were recalculated after exclusion of an outlier data point, believed to be an erroneous case report form entry. Before exclusion of the outlier, the mean liraglutide dose at week 26 was 1.8 mg; the recalculated mean after exclusion of the outlier was 1.7 mg. Degludec, insulin degludec; FPG, fasting plasma glucose; IDegLira, insulin degludec/liraglutide; HbA1c, glycated hemoglobin; U, units.

In the Results section, the author would like to revise the following text: “Participants receiving IDegLira had a lower liraglutide dose (0.9 mg) at week 26 versus those receiving liraglutide (1.8 mg) (Figure 2E)” to “Participants receiving IDegLira had a lower liraglutide dose (0.9 mg) at week 26 versus those receiving liraglutide (1.7 mg) (Figure 2E).”

The section should read:

## 3.5 | Insulin and liraglutide dose

After 26 weeks, participants receiving IDegLira had a statistically significantly lower mean daily insulin dose (24.5 U) versus degludec‐treated participants (30.3 U) (ETD −5.49 U; 95% CI −7.77, −3.21; *P* < 0.0001; Figure 2D). The lower insulin doses (U/kg) with IDegLira were consistent when body weight was accounted for (**Supplementary Table 3**). Participants receiving IDegLira had a lower liraglutide dose (0.9 mg) at week 26 versus those receiving liraglutide (1.7 mg) (Figure 2E).

In Figure 4 caption, the value ‘HbA1c ≤48.0 mmoL/moL’ has been updated to ‘HbA1c ≤47.5 mmoL/moL’. The revised Figure 4 caption is shown below:

**FIGURE 4** Responder endpoints for (A) HbA1c <53.0 mmoL/moL, and (B) HbA1c ≤47.5 mmoL/moL, and composite endpoints for reaching these targets without weight gain and/or without treatment‐emergent severe or confirmed hypoglycemic episodes^†^ (full analysis set) ^†^Treatment‐emergent severe or BG‐confirmed hypoglycemic episodes during the final 12 weeks of treatment. Percentages are observed data. Missing values were imputed using last observation carried forward. Analysis after 26 weeks of treatment based on a logistic regression model with treatment and previous oral antidiabetic treatment as fixed factors. The covariate for: ‘Responder for HbA1c <7.0% and for HbA1c ≤6.5%’, and for ‘HbA1c responder endpoints without hypoglycemic episodes’ was baseline HbA1c; covariates for: ‘HbA1c responder endpoints without weight gain’, and for ‘HbA1c responder endpoints without hypoglycemic episodes and weight gain’ were baseline HbA1c and body weight. BG, blood glucose; CI, confidence interval; degludec, insulin degludec; IDegLira, insulin degludec/liraglutide.

The authors apologize for the errors and any inconvenience these may have caused.

