# Supplementary material for: DUAL I China: Improved glycemic control with IDegLira versus its individual components in a randomized trial with Chinese participants with type 2 diabetes uncontrolled on oral antidiabetic drugs
Source: J Diabetes. 2022 Jun 28;14(6):401–13. doi: 10.1111/1753-0407.13286 (PMC9366571; doi:10.1111/1753-0407.13286)
Supplement: Supplementary file 1 — Appendix S1 Supporting Information [file JDB-14-401-s001.docx]

**SUPPLEMENTARY MATERIAL for:**

**DUAL I China: improved glycemic control with IDegLira versus its individual components in a randomized trial with Chinese participants with type 2 diabetes uncontrolled on oral antidiabetic drugs**

**Supplementary Table 1.** Inclusion and exclusion criteria

| **Inclusion criteria (for an eligible participant, all must be answered ‘yes’)** |
| --- |
| Informed consent obtained before any trial-related activities. Trial-related activities are any procedures that are carried out as part of the trial, including activities to determine suitability for the trial |
| Male or female, age ≥18 years at the time of signing informed consent |
| Type 2 diabetes mellitus (clinically diagnosed) |
| HbA1c 7–10 % (both inclusive) by central laboratory analysis, with the aim of a median of 8.3%. When ~50% of the randomized participants have an HbA1c >8.3%, the remaining participants randomized must have an HbA1c ≤8.3%; or when ~50% of the randomized participants have an HbA1c ≤8.3%, the remaining participants randomized must have an HbA1c >8.3% |
| Current treatment for at least 90 calendar days prior to screening with metformin ± one other OAD: AGI, SU, glinides or TZD. For ≥60 calendar days prior to screening participants should be on a stable dose of:   - Metformin (≥1500 mg or max. tolerated dose) or - Metformin (≥1500 mg or max. tolerated dose) and SU (≥ half of the max. approved dose according to local label) or - Metformin (≥1500 mg or max. tolerated dose) and glinides (≥ half of the max. approved dose according to local label) or - Metformin (≥1500 mg or max. tolerated dose) and AGI (≥ half of the max. approved dose according to local label) or - Metformin (≥1500 mg or max. tolerated dose) and TZD (≥ half of the max. approved dose according to local label) |
| Body mass index ≤40 kg/m^2^ |
| Able and willing to adhere to the protocol including performing self-monitoring of plasma glucose profiles, keeping a trial diary, and using a pre-filled pen device |
| **Exclusion criteria (for an eligible participant, all must be answered ‘no’)** |
| Known or suspected hypersensitivity to trial products or related products |
| Previous participation in this trial. Participation is defined as informed consent |
| Female who is pregnant, breastfeeding or intends to become pregnant or is of childbearing potential and not using adequate contraceptive methods (sterilization, intrauterine device, oral contraceptives, or barrier methods) |
| Receipt of any investigational medicinal product within 30 calendar days prior to visit 1 |
| Current use of any antidiabetic drug (except for metformin, AGI, SU, glinide, or TZD) or anticipated change in concomitant medication, that in the investigator’s opinion could interfere with glucose level (eg, systemic corticosteroids) |
| Use of non-herbal Chinese medicine or other non-herbal local medicine with unknown/unspecified content within 90 calendar days prior to screening. Herbal traditional Chinese medicine or other local herbal medicines may, at the investigator’s discretion, be continued throughout the trial |
| Treatment with insulin (except for short-term treatment at the discretion of the investigator) |
| Treatment with GLP-1 receptor agonists or DPP-4 inhibitors within 90 calendar days prior to screening |
| Impaired liver function, defined as alanine aminotransferase ≥2.5 times upper normal range |
| Impaired renal function defined as serum-creatinine ≥133 μmol/L for males and ≥125 μmol/L for females, or as defined according to local contraindications for metformin |
| Screening calcitonin ≥50 ng/L |
| Personal or family history of MTC or MEN2 |
| Cardiac disorder defined as: congestive heart failure (NYHA class III-IV), diagnosis of unstable angina pectoris, cerebral stroke, and/or myocardial infarction within the last 12 months prior to screening and/or planned coronary, carotid, or peripheral artery revascularization procedures |
| Severe uncontrolled treated or untreated hypertension (systolic blood pressure ≥180 mmHg or diastolic blood pressure ≥100 mmHg |
| Proliferative retinopathy or maculopathy (macular edema), requiring acute treatment |
| Participant with a clinically significant, active (during the past 12 months) disease of the gastrointestinal, pulmonary, neurological, genitourinary, or hematological system (except for conditions associated with T2DM) that in the opinion of the investigator may confound the results of the trial or pose additional risk in administering trial drug |
| Mental incapacity, unwillingness, or language barrier precluding adequate understanding of the trial procedure or cooperation with the trial site personnel |
| Known or suspected abuse of alcohol or narcotics |
| History of pancreatitis (acute or chronic) |
| Suffer from a life-threatening disease including malignant neoplasms and medical malignant neoplasms within the last 5 years (except basal and squamous cell skin cancer) |

NYHA, New York Heart Association classification; OAD, oral antidiabetic drug; AGI, alpha glucosidase inhibitors; SU, sulfonylurea; TZD, thiazolidinedione; HbA1c, glycated hemoglobin; GLP-1, glucagon-like peptide 1; DPP-4, dipeptidyl peptidase 4; ALT, alanine aminotransferase; MTC, medullary thyroid carcinoma; MEN2, multiple endocrine neoplasia type 2; mmHg, millimeters of mercury; T2DM, type 2 diabetes mellitus.

**Supplementary Table 2.** Insulin titration algorithm

| **Mean of three pre-breakfast SMPG (fasting)** | | **Twice-weekly dose adjustments for either IDegLira or degludec** |
| --- | --- | --- |
| **mmol/L** | **mg/dL** | **Units/dose steps** |
| <4.0 | <72 | –2 |
| 4.0–5.0 | 72–90 | 0 |
| >5.0 | >90 | +2 |

Degludec, insulin degludec; IDegLira, insulin degludec/liraglutide; SMPG, self-measured plasma glucose.

**Supplementary Table 3.** Mean actual daily total insulin dose (U/kg) by treatment week

| **Visit** |  | **IDegLira (n = 358)** | **Degludec**  **(n = 175)** |
| --- | --- | --- | --- |
| Visit 3 (week 1) | n | 356 | 174 |
|  | Mean (SD) | 0.16 (0.03) | 0.16 (0.03) |
| Visit 28 (week 26; LOCF) | n | 356 | 174 |
|  | Mean (SD) | 0.33 (0.14) | 0.40 (0.16) |

Degludec, insulin degludec; IDegLira, insulin degludec/liraglutide; LOCF, last observation carried forward; SD, standard deviation.

**Supplementary Table 4.** Mean change from baseline to week 26 in vital signs and treatment comparison

| **Vital sign (baseline)** | **IDegLira**  **(n = 358)** | **Degludec**  **(n = 175)** | **Liraglutide**  **(n = 180)** |
| --- | --- | --- | --- |
| Blood pressure, mmHg, mean (SD) |  |  |  |
| Systolic blood pressure | 128.8 (14.8) | 128.4 (14.3) | 128.0 (14.3) |
| Diastolic blood pressure | 80.0 (9.6) | 79.6 (8.9) | 80.0 (8.6) |
| Heart rate, bpm, mean (SD) | 76.9 (9.1) | 77.3 (10.2) | 77.6 (9.6) |
| **Vital sign (change from baseline)** |  |  |  |
| Blood pressure, mmHg, mean (SD)  Systolic blood pressure  Diastolic blood pressure | −3.4 (12.5) –0.4 (8.2) | –1.2 (11.5)  −0.8 (8.0) | –3.3 (13.6)  −0.1 (8.1) |
| Heart rate, bpm (mean, SD) | 4.3 (8.6) | –0.2 (8.5) | 5.1 (9.8) |
| **Statistical analysis** |  | **IDegLira–degludec ETD (95% CI), *P*-value** | **IDegLira–liraglutide ETD (95% CI), *P*-value** |
| Blood pressure, mmHg  Systolic blood pressure  Diastolic blood pressure | - | –2.04 (–3.95;–0.13), *P* = .0359  0.59 (–0.68;1.86), *P* = .3636 | 0.32 (–1.58;2.22), *P*= .7414  –0.25 (–1.52;1.03), *P*= .7049 |
| Heart rate, bpm | - | 4.29 (2.89;5.69), *P* < .0001 | –1.16 (–2.56;0.24), *P* = .1042 |

*P*-value was two-sided *P*-value for test of no difference. No correction for multiplicity. The response and change from baseline in response after 26 weeks were analyzed using an ANCOVA model with treatment and previous OAD treatment as fixed factors, and corresponding baseline value as covariate. Missing values were imputed by LOCF.

ANCOVA, analysis of covariance; bpm, heartbeats per minute; BP, blood pressure; CI, confidence interval; EDT, estimated treatment difference; LOCF, last observation carried forward; mmHg, millimeters of mercury; N, number of subjects; OAD, oral antidiabetic drug; SD, standard deviation.

**Supplementary Table 5.** Geometric mean of fasting lipid profile week 0 and week 26 (full analysis set)

| **Endpoints** | **IDegLira** | | **Degludec** | | **Liraglutide** | | **IDegLira versus degludec** | **IDegLira versus liraglutide** |
| --- | --- | --- | --- | --- | --- | --- | --- | --- |
|  | **Week 0** | **Week 26** | **Week 0** | **Week 26** | **Week 0** | **Week 26** |  |  |
| Total cholesterol, mmol/L, geom.mean (CV%) | 4.48 (22.1) | 4.22 (21.6) | 4.33 (20.1) | 4.28 (21.7) | 4.54 (21.2) | 4.40 (21.9) | ETR 0.97  95% CI 0.94;0.99  *P* = .0186 | ETR 0.97  95% CI 0.94;1.00  *P*= .0348 |
| HDL cholesterol, mmol/L, geom.mean (CV%) | 1.12 (25.2) | 1.14 (26.4) | 1.12 (25.3) | 1.15 (26.6) | 1.16 (25.1) | 1.17 (25.2) | ETR 0.99  95% CI 0.96;1.02  *P* = .4444 | ETR 1.00  95% CI 0.97;1.02  *P* = .7751 |
| LDL cholesterol, mmol/L, geom.mean (CV%) | 2.37 (36.5) | 2.18 (37.6) | 2.22 (42.7) | 2.22 (48.3) | 2.37 (38.0) | 2.27 (37.7) | ETR 0.94  95% CI 0.89;0.99  *P* = .0276 | ETR 0.96  95% CI 0.90;1.01  *P* = .1153 |
| VLDL cholesterol, mmol/L, geom.mean (CV%) | 0.78 (64.0) | 0.71 (57.9) | 0.78 (54.2) | 0.66 (60.3) | 0.83 (51.6) | 0.77 (55.3) | ETR 1.07  95% CI 0.99;1.15  *P* = .0730 | ETR 0.95  95% CI 0.88;1.02  *P*= .1547 |
| Triglycerides, mmol/L, geom.mean (CV%) | 1.78 (73.5) | 1.58 (64.6) | 1.78 (64.7) | 1.47 (66.7) | 1.89 (60.2) | 1.73 (60.6) | ETR 1.07  95% CI 0.99;1.16  *P* = .0720 | ETR 0.95  95% CI 0.88;1.02  *P* = .1652 |
| Free fatty acids, mmol/L, geom.mean (CV%) | 0.45 (43.0) | 0.25 (63.3) | 0.47 (39.8) | 0.24 (75.1) | 0.45 (44.8) | 0.37 (61.6) | ETR 1.09  95% CI 0.98;1.21  *P* = .1138 | ETR 0.67  95% CI 0.61;0.75  *P* < .0001 |

*P*-value was two-sided *P*-value for test of no difference. No correction for multiplicity. The log-transformed response after 26 weeks was analyzed using an ANCOVA model with treatment and previous OAD treatment as fixed factors, and corresponding log-transformed baseline value as covariate. Missing values were imputed by LOCF.

ANCOVA, analysis of covariance; CI, confidence interval; CV, coefficient of variation; degludec, insulin degludec; ETR, estimated treatment ratio; geom.mean, geometric mean; IDegLira, insulin degludec/liraglutide; HDL, high-density lipoprotein; LDL, low-density lipoprotein; LOCF, last observation carried forward; OAD, oral antidiabetic drug; VLDL, very low-density lipoprotein.

**Supplementary Figure 1.** Participant disposition


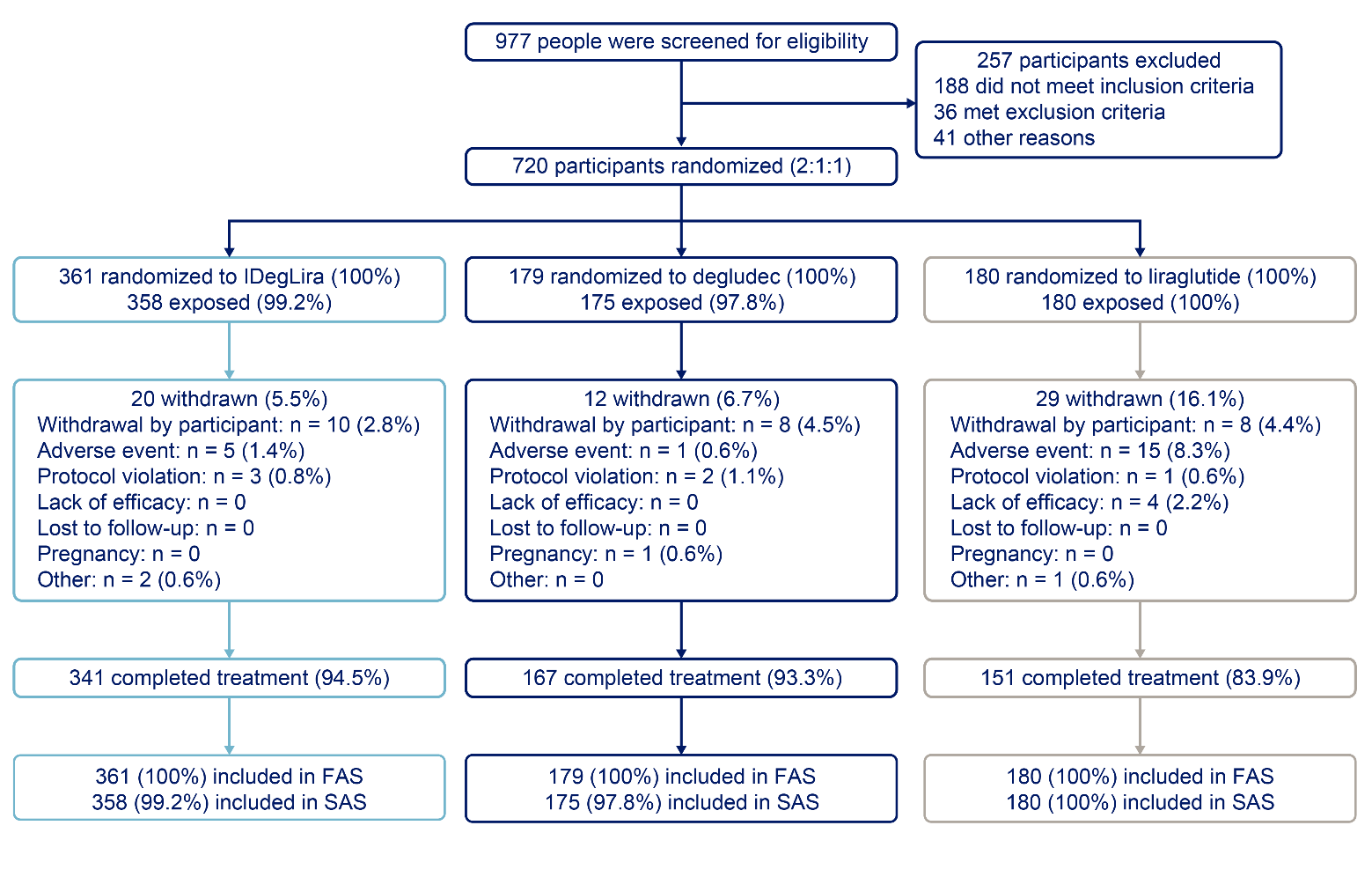


FAS, full analysis set; degludec, insulin degludec; IDegLira, insulin degludec/liraglutide; SAS, safety analysis set.
